# Mineralized Collagen/Polylactic Acid Composite Scaffolds for Load-Bearing Bone Regeneration in a Developmental Model

**DOI:** 10.3390/polym15204194

**Published:** 2023-10-23

**Authors:** Wenbo Zhu, Wenjing Li, Mengxuan Yao, Yan Wang, Wei Zhang, Chao Li, Xiumei Wang, Wei Chen, Hongzhi Lv

**Affiliations:** 1 Department of Orthopaedic Surgery, Hebei Medical University Third Hospital, No. 139 Ziqiang Road, Shijiazhuang 050051, China; spinezwb1917@163.com (W.Z.); m17865583127@163.com (W.L.); mengxuanyao@126.com (M.Y.); 18810310994@163.com (Y.W.); lichao1997@hotmail.com (C.L.); 2 Key Laboratory of Biomechanics of Hebei Province, Orthopaedic Research Institution of Hebei Province, No. 139 Ziqiang Road, Shijiazhuang 050051, China; 3 National Health Commission Key Laboratory of Intelligent Orthopaedic Equipment, Hebei Medical University Third Hospital, No. 139 Ziqiang Road, Shijiazhuang 050051, China; 4 Department of Pathology, Hebei Medical University, No. 361 Zhongshan Road, Shijiazhuang 050017, China; zwbL201309@126.com; 5 State Key Laboratory of New Ceramics and Fine Processing, School of Materials Science and Engineering, Tsinghua University, No. 30 Shuangqing Road, Beijing 100084, China; wxm@mail.tsinghua.edu.cn

**Keywords:** mineralized collagen, polylactic acid, children, bone remodeling, bone regeneration

## Abstract

Repairing load-bearing bone defects in children remains a big clinical challenge. Mineralized collagen (MC) can effectively simulate natural bone composition and hierarchical structure and has a good biocompatibility and bone conductivity. Polylactic acid (PLA) is regarded as a gold material because of its mechanical properties and degradability. In this study, we prepare MC/PLA composite scaffolds via in situ mineralization and freeze-drying. Cell, characterization, and animal experiments compare and evaluate the biomimetic properties and repair effects of the MC/PLA scaffolds. Phalloidin and DAPI staining results show that the MC/PLA scaffolds are not cytotoxic. CCK-8 and scratch experiments prove that the scaffolds are superior to MC and hydroxyapatite (HA)/PLA scaffolds in promoting cell proliferation and migration. The surface and interior of the MC/PLA scaffolds exhibit rich interconnected pore structures with a porosity of ≥70%. The XRD patterns are typical HA waveforms. X-ray, micro-CT, and H&E staining reveal that the defect boundary disappears, new bone tissue grows into MC/PLA scaffolds in a large area, and the scaffolds are degraded after six months of implantation. The MC/PLA composite scaffold has a pore structure and composition similar to cancellous bone, with a good biocompatibility and bone regeneration ability.

## 1. Introduction

In recent years, various absorbable materials have been developed to repair bone defects, and significant progress has been made in artificial bone replacement [1,2]. As most studies have focused on adult bones, there are no suitable materials for repairing load-bearing bone defects in children [3,4]. Children are in the peak period of growth and development, and their bones continue to grow. Therefore, bone repair materials suitable for children need to induce and maintain the process of bone healing and dynamic structural characteristics to adapt to the continuous growth of bones [5]. The materials should also exhibit the appropriate mechanical properties. The implant material should have an elastic modulus similar to that of bone tissue to avoid stress shielding and provide mechanical support for the defect until bone tissue regeneration [6,7].

Biomimetic mineralized collagen (MC) comprises type I collagen (Col I) and hydroxyapatite (HA). It is a bone repair material that works on the principle of in situ biomimetic mineralization and molecular self-assembly technology, which can simulate the composition and hierarchical structure of natural bones. It exhibits a good biocompatibility, bone conductivity, and biological activity, provides a suitable cellular microenvironment for bone regeneration, and has applications in bone defect repair [8,9,10,11,12]. However, the mechanical properties of simple MC scaffolds are limited and need to meet the high mechanical strength and dynamic property requirements for bone repair materials [13]. 

Polylactic acid (PLA) is a polymer derived from the polymerization of lactic acid produced by biological fermentation. A single lactic acid molecule has a hydroxyl group and a carboxyl group. Multiple lactic acid molecules are together, and the hydroxyl group is dehydrated with the carboxyl group of other molecules. The carboxyl group is dehydrated with the hydroxyl group of other molecules to form polylactic acid [14]. It is a natural and eco-friendly aliphatic polyester obtained from various sources. It exhibits excellent mechanical properties, biocompatibility, and plasticity and is a good biodegradable regenerative material. It is widely used in biodegradable sutures, bone fixation devices, surgical implant materials, and drug delivery systems [15,16,17,18,19,20,21,22]. Many studies have shown that PLA can improve implant materials’ mechanical, osteogenic, and chondrogenic properties [23,24,25,26,27]. 

In this study, we introduced PLA as a composite material to fabricate MC/PLA artificial bone repair scaffolds. We also evaluated their physicochemical properties via biological characterization and biocompatibility in vitro via cell experiments. Finally, we established a femur defect model in developing sheep, implanted the scaffolds in vivo, and evaluated their effect on bone defect repair via imaging and histological analyses. 

## 2. Materials and Methods

### 2.1. Fabrication of MC Power and the MC/PLA Composite Scaffold

MC is a biomimetic material with high osteogenic activity, synthesized using in situ mineralization technology for an initial theoretical Col I/HA mass ratio from 10/90 to 20/80. The synthesis of the scaffolds started with the dissolution of the Col I sponge (purity > 99%; Kaolisen, Beijing, China) in acetic acid and stirring with magnetic stirrers (MS3; Quan, Beijing, China) for more than 10 h until the Col I was completely dissolved. The solution was then slowly dropped into a calcium chloride and phosphate solution and vigorously stirred for 2 h. The resulting solution was added dropwise to a sodium hydroxide solution (0.5 mol/L) to adjust the pH to 10, and the mixture was stirred for 10 h. The mineralized suspension was centrifuged at 8000 rpm for 3 min, and the precipitate was removed and cleaned. The centrifugation and cleaning were repeated until the pH of the supernatant reached 7. The precipitate was pre-frozen at −20 °C for 24 h, vacuum lyophilized at −50 °C for 48 h in a freeze-drying machine (FD-1-50; Boyikang, Beijing, China), and ground into powder. PLA (biomedical grade; DaiGang, Beijing, China) was dissolved in 1, 4-dioxane and stirred for 1 h to form a homogeneous, stable liquid mixture. The acquired MC powder was added under continuous stirring at a mass ratio of MC to PLA (45:55 to 60:40). The resulting slurry was molded into a sheet mold to fabricate the scaffold for the cell experiments; a cylindrical mold was used for the characterization and animal experiments. The final interconnected porosity of the scaffolds was determined by freeze-drying. Briefly, the scaffolds were pre-frozen at −20 °C for 48 h and subjected to vacuum lyophilization at −50 °C for 72 h. The compressive strength of the scaffold was 0.8–1.2 MPa, the porosity was ≥70%, and the network structure was porous. The scaffolds were then completely immersed in anhydrous ethanol, cleaned with ultrasound to remove the mixed solvent, and stored at room temperature. After fabrication, all the scaffolds used for the cell or animal experiments were sterilized via ^60^Co irradiation at 15–25 kGy.

### 2.2. Characterization of MC Power and Natural Bone

A phase analysis of the MC power was conducted using an X-ray diffraction (XRD) diffractometer (SmartLab; Rigaku Corporation, Tokyo, Japan). The detailed morphology and microstructure of the MC powder were observed using transmission electron microscopy (Aojing, Beijing, China). We also compared the analysis results of the scaffold with those of natural bone tissue.

### 2.3. Cell Culture

This study used bone marrow mesenchymal stem cells (BMSCs; Cell Bank, Chinese Academy of Sciences, Shanghai, China) and mouse pericranial bone cells (MC3T3-E1; Cell Bank, Chinese Academy of Sciences, Shanghai, China). The BMSCs were cultured in α-MEM with 10% (*v*/*v*) fetal bovine serum (FBS; Gibco, GrandIsland, NY, USA) and 1% (*v*/*v*) penicillin/streptomycin (PS; Gibco, GrandIsland, NY, USA). The MC3T3-E1 cells were cultured in α-MEM with 10% FBS and incubated at 37 °C with 5% CO_2_. The prepared scaffolds were immersed in alpha-minimum necessary medium (α-MEM; Gibco, GrandIsland, NY, USA) and soaked at 37 °C for 72 h; the mass volume ratio of the sample to the medium was 0.05 g/mL, and the extracts were collected and stored at 4 °C. Simultaneously, the HA/PLA scaffold (Aojing, Beijing, China) and MC were collected using the abovementioned method.

### 2.4. Phalloidin and 4′,6-Diamidino-2-phenylindole Dye (DAPI) Staining

Extracts from different materials were used to grow the MC3T3-E1 cells, and α-MEM was used as the blank control. The filamentous actin and cell nuclei of the MC3T3-E1 cells were stained with phalloidin and DAPI, respectively, at 1 and 5 d. The cytoskeletal structure was observed using an Olympus fluorescence microscope (Olympus Bx 53; Tokyo, Japan).

### 2.5. Cell Proliferation

A cell counting kit (CCK)-8 (Dojindo, Kumamoto, Japan) was used to assess the cell proliferation. The MC3T3-E1 cells were plated in 96-well plates at a 5 × 10^5^ cells/well density. After 24 h of culturing at 37 °C and 5% CO_2_, the medium was replaced with MC, MC/PLA, and HA/PLA extracts, and a blank control group was set up without treatment. After incubation for 1, 3, and 5 d, 10 μL of CCK-8 reagent was added to each well and incubated for 2 h. The optical density was measured at 450 nm using enzyme markers, and cell proliferation was compared among the groups.

### 2.6. Cell Migration

The effects of different materials on the migration ability of the BMSCs were compared using scratch-healing experiments. The BMSCs were inoculated into 6-well plates and cultured to 90% confluency. A scratch was made using the tip of a 1 mL sterile pipette, extracts of different materials were added, and a blank control group was set up. Wound images were collected from each group at 0, 24, 36, and 48 h, and the healing rate was calculated using the ImageJ software (version 1.8.0; National Institutes of Health, Bethesda, MD, USA).

### 2.7. Characterization of the MC/PLA Scaffold

The pore morphology, size, and distribution of the MC/PLA scaffolds were observed using SEM (Regular8100; Hitachi, Tokyo, Japan) after gold spraying. Simultaneously, the calcium–phosphorus ratio of the scaffold was measured using an EDS detector. The transmitting voltage was 15 kV and the working distance was 5–6 mm. An XRD analysis of the MC/PLA scaffold was conducted by grinding the scaffold into fine particles with a particle size of < 40 μm. The crystal phase of the scaffold was determined by comparing the diffraction patterns with those of the standard PDF card. The diffraction conditions were as follows: Cu Kα radiation was generated at 40 kV and 40 mA, and spectra were recorded in the 2θ range of 20–60° with a scanning speed of 1.8 s/step and a step size of 0.02°. The grain size was calculated using the Jade6.5 software (MDI, Los Angeles, CA, USA). The scaffold’s porosity was measured using an automated mercury injection instrument (AutoPoreIV9500; Micromeritics, Atlanta, GA, USA). Briefly, the scaffold was trimmed into a cube (10 mm^3^ × 10 mm^3^ × 10 mm^3^), dried, weighed, and placed in a dilatometer for complete sealing. The dilatometer was successively placed in low- and high-pressure stations for a porosity analysis. The compressive strength of the MC/PLA scaffold was measured using a universal mechanical tester (AG-IC; Shimadzu, Kyoto, Japan) and compared with that of natural bone tissue. The pressure load range was 0–250 N.

### 2.8. In Vivo Animal Experiments, the MC/PLA Scaffold

In this experiment, young developing animals, healthy sheep aged 3 months and weighing 10–25 kg, were selected to prepare bone defects with a length of 2 cm in the middle part of the femur of each sheep to construct an animal model of long bone defects in the developing stage. To assess the MC/PLA scaffold integration and subsequent bone regeneration, sterilized MC/PLA scaffolds were implanted at the defect site, while, in the control group, no implants were implanted and fixed with steel plates. Femur samples were collected 1, 3, and 6 months after implantation. X-ray and micro-computed tomography (micro-CT) were performed on the bone defects, and the bone density information in the defect area was collected to compare the coverage of new bone tissue between the two groups. A histological evaluation of the femur samples was performed. Sections (5 μm thick) were prepared after the decalcification, washing, dehydration, and embedding of the femur. The sections were stained with hematoxylin and eosin (H&E), methylene acid fuchsin, and Goldner’s trichrome and observed under an optical microscope (IX81; OLYMPUS, Tokyo, Japan).

### 2.9. Statistical Analysis

The results are expressed as the average ± standard error. Data from the MC, MC/PLA, HA/PLA, and blank control groups were compared using a one-way analysis of variance, followed by the least significant difference post hoc test. Statistical analyses were conducted using the SPSS software (version 23.0; SPSS, Chicago, IL, USA), with statistical significance set at *p* < 0.05.

## 3. Results and Discussion

### 3.1. Comparison of the Characteristics of MC and Natural Bone

**Figure 1:**
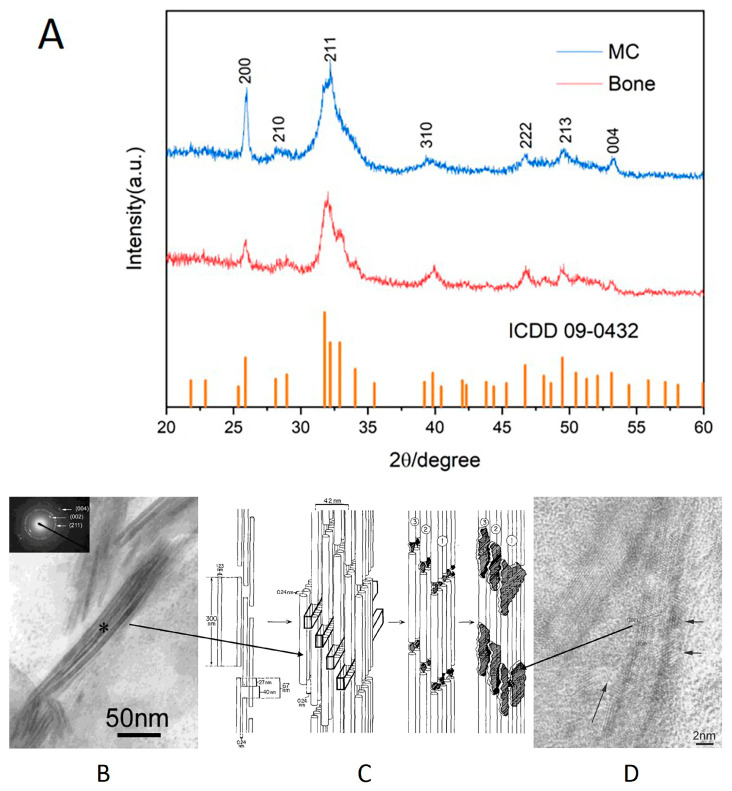
Comparison between MC and natural bone. (**A**) XRD of MC and natural bone; (**B**) TEM and selective electron diffraction analysis of MC fibers, ∗: the center of the selected electron diffraction pattern area [28]. Reprinted (adapted) with permission from {COMPLETE REFERENCE CITATION}. Copyright {2003} American Chemical Society. (**C**) Schematic diagram of MC microstructure [29], Copyright © 1993 by Academic Press, Inc. (**D**) The C-axis of HA was parallel to the long axis of the collagen fibers, consistent with the natural bone structure [28]. Reprinted (adapted) with permission from {COMPLETE REFERENCE CITATION}. Copyright {2003} American Chemical Society.

The MC bone powder was synthesized through an innovative, biologically inspired synthetic process, which recapitulated the main steps of bone biomineralization and self-assembly. The XRD patterns revealed that the shape and distribution of the diffraction peaks of the MC were consistent with those of human bone tissue (Figure 1A). According to an electron diffraction analysis (Figure 1B–D), the HA crystal grew in the interstitial space of collagen fibers, and its c-axis preferred orientation was parallel to the long axis of the collagen fibers, which was similar to the crystal composition and nanostructure of natural human bone. The composition and structure of the MC were confirmed by the XRD and electron diffraction analyses at the molecular level, which indicated that MC can simulate natural bone well in terms of its composition and hierarchical structure.

### 3.2. Comparison of the Cell Compatibility of MC/PLA, HA/PLA, and MC Scaffolds

**Figure 2:**
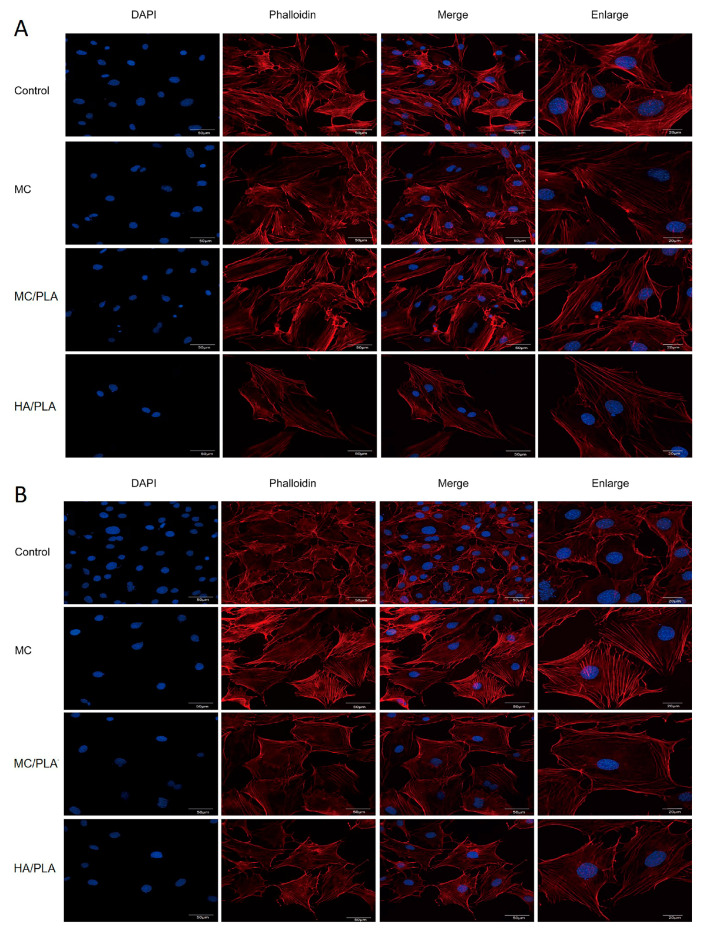

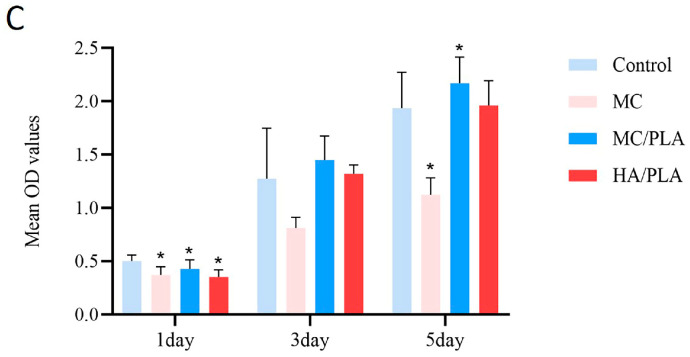
Effects of different materials on cell activity and cell proliferation. Fluorescence images of cytoskeleton-stained MC3T3-E1 cells cultured in a medium containing different materials (control, MC, MC/PLA, and HA/PLA) for 1 d (**A**) and 5 d (**B**), (**C**) proliferation of MC3T3-E1 cells in medium containing different materials, measured by CCK-8 (* *p* < 0.05 compared with the control group).

PLA is one of the most common degradable materials with good osteogenic activity, mechanical properties, and plasticity. We introduced it into the MC and HA, and the composite scaffolds were prepared via freeze-drying to explore the feasibility of its application in the repair and treatment of bearing bone defects in the development stage. The stability, degradability, and biocompatibility of bone defect repair materials are essential for their clinical application, and cell compatibility experiments are an effective method for verifying their biocompatibility [30,31,32,33]. We used MC3T3-E1 cells to verify the effects of the MC/PLA, MC, and HA/PLA scaffolds on cytotoxicity and cell proliferation. The original medium was used as a blank control. The expression of the actin cytoskeleton is an important indicator of cell morphology. Figure 2A,B show the cell morphologies of the materials co-cultured with MC3T3-E1 cells for 1 and 5 d, respectively. Red filamentous cytoskeletal proteins were observed, and most of the nuclei were completely oval, indicating that the cells were very healthy and active. The effects of different scaffold materials on the proliferation of the MC3T3-E1 cells were determined using the CCK-8 assay. As shown in Figure 2C, the OD values of the three materials were lower than that of the blank control group on the first day of culture (*p* < 0.05), and cell proliferation was not obvious. After 3 d of culture, there was a difference in the effect of the three materials on cell proliferation compared to the control group, but this difference was not statistically significant (*p* > 0.05). When the co-culture time was prolonged to 5 d, the OD value of the MC/PLA group was higher than that of the control group (*p* < 0.05), and the proliferation promotion ability was better than that of the MC and HA/PLA groups. The results showed that the MC/PLA scaffold had no toxicity to cell viability and could promote cell proliferation compared to the control, MC, and HA/PLA groups.

**Figure 3:**
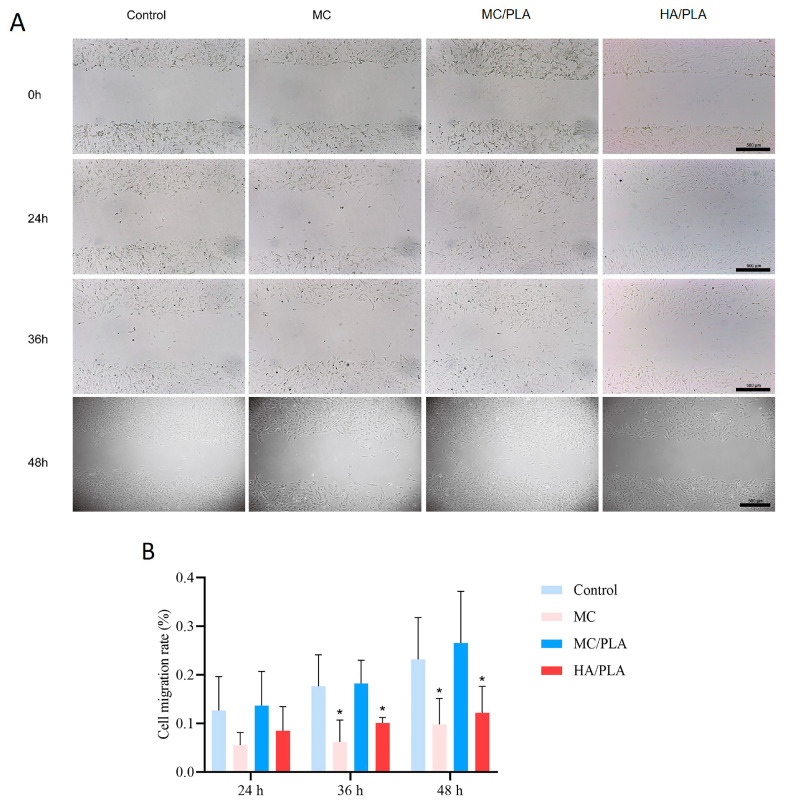
Effects of different materials on cell migration. (**A**) Wound images of BMSCs were collected at 0, 24, 36, and 48 h when cultured in a medium containing different materials (control group, MC, MC/PLA, and HA/PLA) with scratches. (**B**) Scratch healing on medium containing different materials, cell mobility as mean ± standard deviation (* *p* < 0.05 compared with the control group).

A prerequisite for repair is that BMSCs are mobilized from the bone marrow to the injured site through peripheral circulation during healing [34,35]. We compared the effects of MC/PLA, MC, and HA/PLA on the migration and recruitment of BMSCs. As shown in Figure 3, in the scratch-healing experiment, the cell migration rate of the MC/PLA group was higher than that of the MC and HA/PLA groups, which could significantly promote BMSC migration. However, the cell migration rate of the MC/PLA group was slightly higher than that of the control group, but the difference was not statistically significant (*p* > 0.05). MC/PLA materials could significantly promote the migration of cells, which was conducive to the repair of bone defects. 

In vitro cell experiments showed that the MC/PLA composite scaffolds had a good cytocompatibility and met the requirements for tissue engineering. Dewey et al. [36] showed that PLA added to an MC scaffold to form a composite material had a favorable effect on adipose-derived stem cells’ vitality and proliferation ability, similar to this study’s results.

### 3.3. Characteristics and Mechanical Properties of the MC/PLA Scaffold

**Figure 4:**
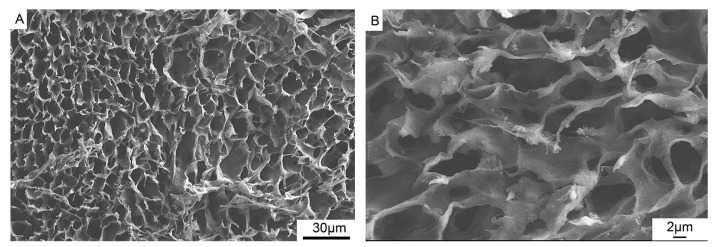
SEM images of the MC/PLA scaffold. (**A**) The surface image and (**B**) enlarged cross-section of (**A**).

**Figure 5:**
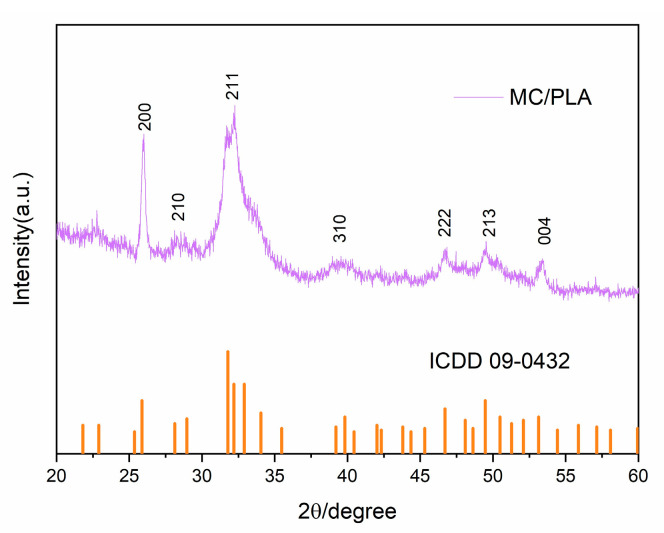
XRD of the MC/PLA scaffold.

Figure 4 shows that the MC/PLA scaffold was a three-dimensional porous material. The SEM images show that the scaffold had an ordered honeycomb structure, and interconnected multi-stage pore structures were present on the surface and interior of the scaffold. The ca/P ratio of the MC/PLA scaffold was 1.67, consistent with that of HA. The XRD pattern of the MC/PLA scaffold (Figure 5) revealed that, compared to the standard card ICDD09-0432 of HA, the diffraction peak distribution of this material was similar to that of HA, and the main peaks were located near 26 and 32°, that is, the characteristic peaks of HA, with no obvious peaks of calcium phosphate and other crystalline substances. Moreover, the material’s grain size was distributed between 20 and 50 nm. As mentioned above, the scaffold contained the HA phase according to the XRD pattern and Ca/P ratio. The addition of PLA did not affect the composition of the HA crystals. 

In addition, the porosity of the MC/PLA composite scaffold was ≥70%, measured using a mercury injection instrument, and the compressive strength of the scaffold was 0.8–1.2 MPa, measured using a mechanical tester. The porosity of the scaffolds (≥70%) showed similar values to those found in cancellous bone (around 30–90%), providing the required space for the retention of nutrients, the growth of blood vessels, and the adhesion and growth of cells, playing an important role in osteogenesis [37,38]. In this study, the compressive strength of the MC/PLA composite scaffold was 0.8–1.2 MPa. Compared to MC, the mechanical properties of the MC/PLA composite scaffold were enhanced, but they were still lower than the compressive strength of the spongy bone (2–12 MPa) and cortical bone (170–230 MPa) [39,40]. Therefore, the mechanical properties of the composite scaffold must be further improved.

The characterization of the MC/PLA composite scaffold indicated that the composite scaffold was a bionic bone repair material with a good structural composition and physical and chemical properties.

### 3.4. In Vivo Animal Experiments

The MC/PLA composite scaffold was implanted into the critical femoral defect model of a 3-month-old sheep, and the regeneration and repair ability of the material for weight-bearing bone defects was evaluated. Unfortunately, the sheep in the control group broke the steel plate 2 weeks after the operation, so we only obtained the experimental group’s results.

**Figure 6:**
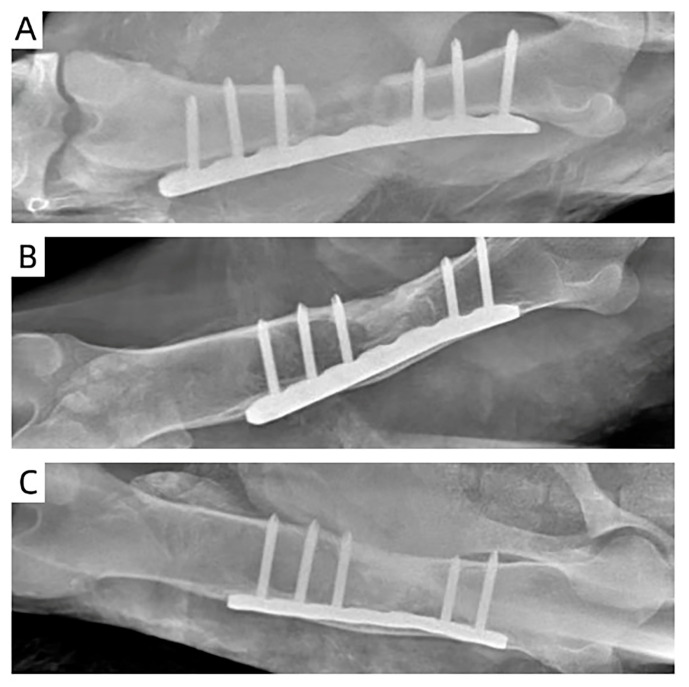
X-ray images of the femur defect at 1 (**A**), 3 (**B**), and 6 (**C**) months after the MC/PLA scaffold implantation in 3-month-old sheep.

**Figure 7:**
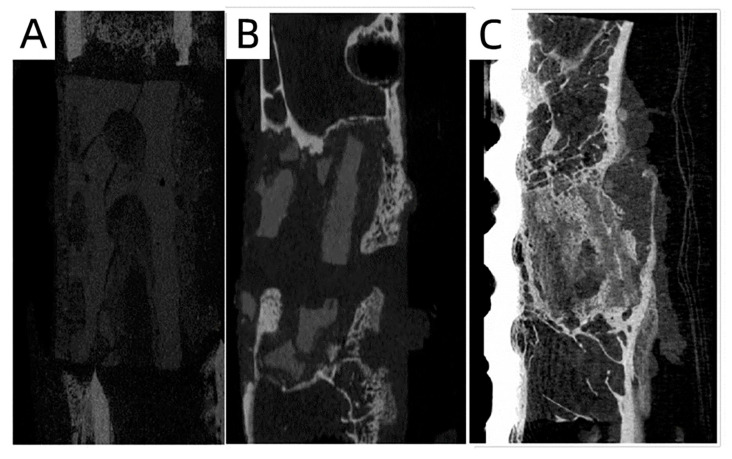
Micro-CT images of the femur defect at 1 (**A**), 3 (**B**), and 6 (**C**) months after the MC/PLA scaffold implantation in 3-month-old sheep.

Figure 6 shows X-ray images of a 3-month-old sheep after the implantation of the MC/PLA scaffold at the site of the bone defect. The sheep grew healthily without rejection one month after the scaffold implantation, and the scaffold was fixed well. Three months after the implantation, the formation of bone tissue was obvious; there were bone bridges at both ends of the defect, the boundary between the bone defect area and the original bone became blurred, and a large area of new bone tissue had replaced the scaffold. The bone tissue grew further at six months, covering almost the entire area of the bone defect. Figure 7 shows micro-CT images obtained after the scaffold implantation. The implant material remained intact and new bone tissue formed after 1 month. Compared to those at 1 month, the images obtained at 3 months showed more obvious bone tissue repair and the scaffolds began to degrade. After 6 months of repair, the scaffold degraded and a large amount of new bone tissue grew into the defect. 

**Figure 8:**
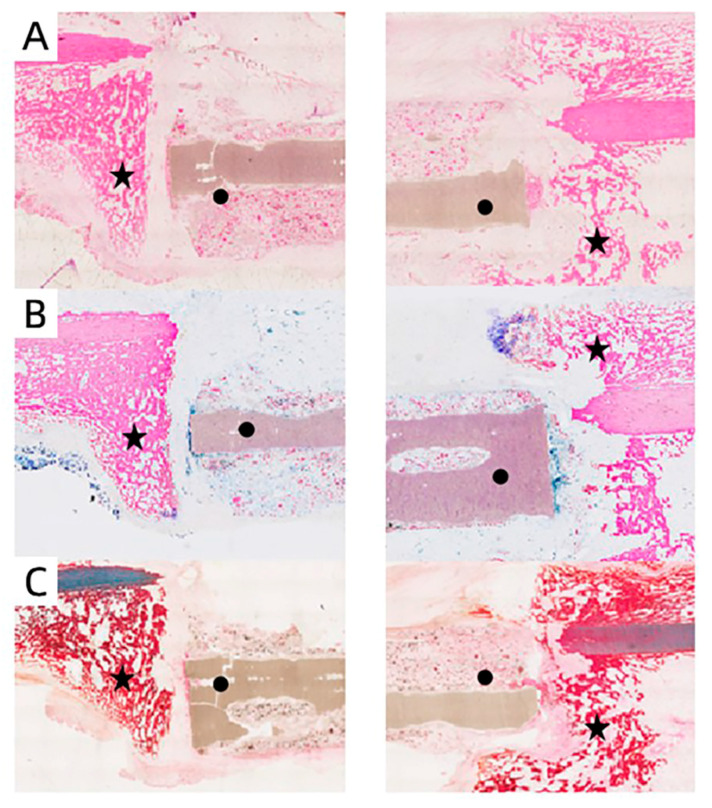
H&E (**A**), methylene acid fuchsin (**B**), and Goldner’s trichrome (**C**) staining of bone defect site 1 month after scaffold implantation. ★: new bone tissue, ●: scaffold.

**Figure 9:**
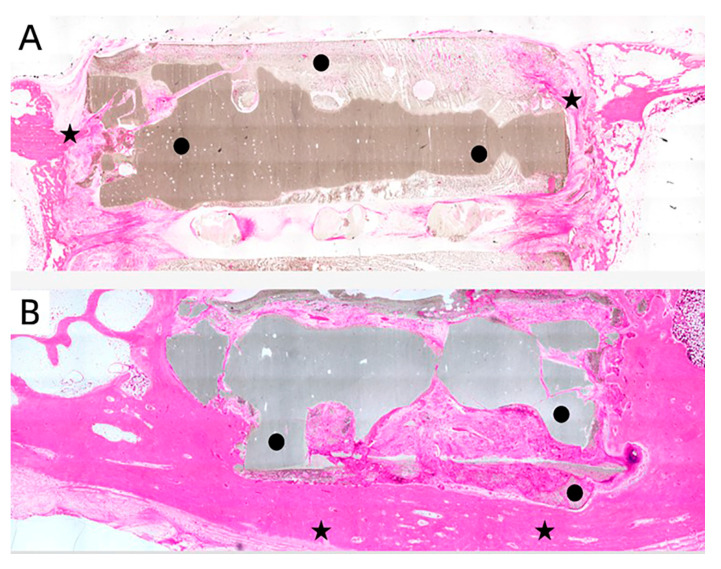
H&E staining of bone defect site at 3 months (**A**) and 6 months (**B**) after scaffold implantation. ★: new bone tissue, ●: scaffold.

To further explore the effect of the MC/PLA composite scaffold on the regeneration and repair of load-bearing bone during development, we performed a histological examination of the site of the bone defects in the sheep. In the three staining images, it can be seen that, one month after the operation, the implant’s contour was still distinguishable, no obvious medullary cavity was observed, and no large-scale material degradation occurred (Figure 8). The appearance of the medullary cavity indicated that the material began to degrade while inducing new bone formation. As shown in Figure 9, extensive infiltration of inflammatory cells and osteoblasts was observed in the H&E-stained images 3 months after the operation. Bone tissue grew on the surface of the material, new bone trabeculae were observed, and the material degraded gradually. Six months after the operation, a wide range of new bone tissue was formed, the new bone trabeculae grew into the scaffold in a large area, and the material degraded significantly. The degradation rate of the scaffolds was in line with the growth rate of sheep femurs during development. 

The MC/PLA composite scaffolds provided a suitable environment for promoting the growth and migration of cells and exhibited long-term in vivo biocompatibility and bone regeneration capacity for repairing bone defects during development, consistent with previous research results [41].

## 4. Conclusions

Preparing materials suitable for bearing bone defects in children has always been a difficult problem in orthopedic biomaterials, especially developing materials with biocompatibility and the appropriate mechanical strength. For bone repair material MC, which conforms to natural bone structure and has good biological activity, it is difficult to solve the problem of its weak mechanical strength due to its inherent structure. PLA is a biopolymer widely used in tissue engineering owing to its high mechanical strength, good biocompatibility, and degradability. In the present work, we investigated a biomimetic composite MC/PLA by introducing PLA. The MC/PLA scaffold was applied to repair critical-sized segmental femur defects in developing sheep to clinically simulate the repair treatment of bearing bone defects in children. In vitro cell experiment results showed that the MC/PLA scaffold had no toxicity to cell viability and could promote cell proliferation compared to the control, MC, and HA/PLA groups. We compared the effects of MC/PLA, MC, and HA/PLA on the migration and recruitment of BMSCs and found that MC/PLA materials could significantly promote the migration of cells, which was conducive to the repair of bone defects. 

Moreover, we further studied the characterization of the MC/PLA scaffolds. The composite material was an interconnected multi-stage pore structure on both the surface and inside. Its porosity (≥70%) showed similar values to those found in cancellous bone (around 30–90%). The XRD pattern was consistent with the HA standard diffraction card, and the calcium–phosphorus ratio was consistent with the element proportion in HA. Through the characterization of the MC/PLA composite scaffolds, it could be seen that the scaffold material had a good porosity and pore structure suitable for bone growth, its composition was consistent with that of natural bone, and the addition of PLA did not affect the composition of the HA crystal. It indicated that the composite scaffold was a bionic bone repair material with a good structural composition and physical and chemical properties. Finally, we established a femur defect model in developing sheep, implanted the MC/PLA scaffolds, and analyzed the scaffolds’ in vivo bone repair and regeneration ability using imaging and histological methods. After the implantation of the scaffolds, the extensive infiltration of inflammatory cells and osteoblasts was observed, and new bone tissue continuously formed on the surface and in the internal pores of the scaffolds. The surrounding bone was continuous, the material was well-connected to the natural bone tissue, and the defect site was repaired over a large area. It is worth noting that, during the period from implantation to 6 months after surgery, the material was degraded continuously with the regeneration of the bone, and the degradation rate of the scaffold adapted to the growth rate of the sheep femur during development.

In this study, we fabricated an MC/PLA composite scaffold as an artificial bone implant material and observed its excellent performance in the repair of bone defects. The scaffold had a suitable porosity, good physical and chemical properties, and a good cytocompatibility. It also promoted the proliferation and migration of BMSCs. Moreover, the stent significantly promoted bone regeneration and repair in developing sheep with femur defects. Therefore, the MC/PLA scaffold can potentially be used to repair bone tissue defects in children.

## Data Availability

The data supporting this study’s findings are available from the corresponding author upon reasonable request.

## References

[1] Bondareva J.V., Dubinin O.N., Kuzminova Y.O., Shpichka A.I., Kosheleva N.V., Lychagin A.V., Shibalova A.A., Pozdnyakov A.A., Akhatov I.S., Timashev P.S. (2022). Biodegradable iron-silicon implants produced by additive manufacturing. Biomed. Mater..

[2] Kumar A., Mir S.M., Aldulijan I., Mahajan A., Anwar A., Leon C.H., Terracciano A., Zhao X., Su T.L., Kalyon D.M. (2021). Load-bearing biodegradable PCL-PGA-beta TCP scaffolds for bone tissue regeneration. J. Biomed Mater. Res. B Appl. Biomater..

[3] Hersh D.S., Anderson H.J., Woodworth G.F., Martin J.E., Khan Y.M. (2021). Bone Flap Resorption in Pediatric Patients Following Autologous Cranioplasty. Oper. Neurosurg..

[4] Jain S., Wang S., Sandoval-Garcia C., Ibrahim G.M., Robinson W.L., Ragheb J. (2021). Autologous Calvarial Bone Remodeling Technique for Small to Medium-Sized Cranial Defects in Young Children: The “Switch-Cranioplasty” Technique. Pediatr. Neurosurg..

[5] Brugmans M.M., Soekhradj-Soechit R.S., van Geemen D., Cox M., Bouten C.V., Baaijens F.P., Driessen-Mol A. (2016). Superior Tissue Evolution in Slow-Degrading Scaffolds for Valvular Tissue Engineering. Tissue Eng. Part A.

[6] Bhuiyan D.B., Middleton J.C., Tannenbaum R., Wick T.M. (2016). Mechanical properties and osteogenic potential of hydroxyapatite-PLGA-collagen biomaterial for bone regeneration. J. Biomater. Sci. Polym. Ed..

[7] Yavari S.A., van der Stok J., Chai Y.C., Wauthle R., Birgani Z.T., Habibovic P., Mulier M., Schrooten J., Weinans H., Zadpoor A.A. (2014). Bone regeneration performance of surface-treated porous titanium. Biomaterials.

[8] Yang L., Kong J., Qiu Z., Shang T., Chen S., Zhao R., Raucci M.G., Yang X., Wu Z. (2020). Mineralized collagen-modified PMMA cement enhances bone integration and reduces fibrous encapsulation in the treatment of lumbar degenerative disc disease. Regen. Biomater..

[9] Gomes A.D., de Oliveira A.A., Houmard M., Nunes E.H. (2021). Gamma sterilization of collagen/hydroxyapatite composites: Validation and radiation effects. Appl Radiat Isot..

[10] Mazzoni E., D'Agostino A., Iaquinta M.R., Bononi I., Trevisiol L., Rotondo J.C., Patergnani S., Giorgi C., Gunson M.J., Arnett G.W. (2020). Hydroxylapatite-collagen hybrid scaffold induces human adipose-derived mesenchymal stem cells to osteogenic differentiation in vitro and bone regrowth in patients. Stem Cells Transl. Med..

[11] Dai Y., Xu J., Han X.-H., Cui F.-Z., Zhang D.-S., Huang S.-Y. (2021). Clinical efficacy of mineralized collagen (MC) versus anorganic bovine bone (Bio-Oss) for immediate implant placement in esthetic area: A single-center retrospective study. BMC Oral Health.

[12] Gönder N., Demir İ.H., Öğümsöğütlü E., Kılınçoğlu V. (2021). Collagen/Nano-hydroxyapatite Composite Scaffold Application with Exchange Reamed Nailing Accelerates Bone Union and Improves Quality of Life in Atrophic Femoral Shaft Nonunions: A Retrospective Comparative Study. Indian J. Orthop..

[13] Ohba S., Shido R., Asahina I. (2021). Application of hydroxyapatite/collagen composite material for maxillary sinus floor augmentation. J. Oral Sci..

[14] Li G., Zhao M., Xu F., Yang B., Li X., Meng X., Teng L., Sun F., Li Y. (2020). Synthesis and Biological Application of Polylactic Acid. Molecules.

[15] Murariu M., Dubois P. (2016). PLA composites: From production to properties. Adv. Drug Deliv. Rev..

[16] Carvalho J.R.G., Conde G., Antonioli M.L., Santana C.H., Littiere T.O., Dias P.P., Chinelatto M.A., Canola P.A., Zara F.J., Ferraz G.C. (2021). Long-Term Evaluation of Poly(lactic acid) (PLA) Implants in a Horse: An Experimental Pilot Study. Molecules.

[17] Ilyas R.A., Zuhri M.Y.M., Aisyah H.A., Asyraf M.R.M., Hassan S.A., Zainudin E.S., Sapuan S.M., Sharma S., Bangar S.P., Jumaidin R. (2022). Natural Fiber-Reinforced Polylactic Acid, Polylactic Acid Blends and Their Composites for Advanced Applications. Polymers.

[18] Lett J.A., Sagadevan S., Léonard E., Fatimah I., Hossain M.A.M., Mohammad F., Al-Lohedan H.A., Paiman S., Alshahateet S.F., Razak S.I.A. (2021). Bone tissue engineering potentials of 3D printed magnesium-hydroxyapatite in polylactic acid composite scaffolds. Artif. Organs.

[19] Joshi D.S., Singhvi M.S., Khire J.M., Gokhale D.V. (2010). Strain improvement of Lactobacillus lactis for D-lactic acid production. Biotechnol. Lett..

[20] Vasir J.K., Labhasetwar V. (2007). Biodegradable nanoparticles for cytosolic delivery of therapeutics. Adv. Drug Deliv. Rev..

[21] Chen S., Guo R., Liang Q., Xiao X. (2021). Multifunctional modified polylactic acid nanofibrous scaffold incorporating sodium alginate microspheres decorated with strontium and black phosphorus for bone tissue engineering. J. Biomater. Sci. Polym. Ed..

[22] Singhvi M.S., Zinjarde S.S., Gokhale D.V. (2019). Polylactic acid: Synthesis and biomedical applications. J. Appl. Microbiol..

[23] Mozdzen L.C., Rodgers R., Banks J.M., Bailey R.C., Harley B.A. (2016). Increasing the strength and bioactivity of collagen scaffolds using customizable arrays of 3D-printed polymer fibers. Acta Biomater..

[24] Ray S., Adelnia H., Ta H.T. (2021). Collagen and the effect of poly-l-lactic acid based materials on its synthesis. Biomater. Sci..

[25] Cui M., Liu L., Guo N., Su R., Ma F. (2015). Preparation, cell compatibility and degradability of collagen-modified poly(lactic acid). Molecules.

[26] Liu S., Zheng Y., Liu R., Tian C. (2020). Preparation and characterization of a novel polylactic acid/hydroxyapatite composite scaffold with biomimetic micro-nanofibrous porous structure. J. Mater. Sci. Mater. Med..

[27] Marycz K., Smieszek A., Targonska S., Walsh S.A., Szustakiewicz K., Wiglusz R.J. (2020). Three dimensional (3D) printed polylactic acid with nano-hydroxyapatite doped with europium(III) ions (nHAp/PLLA@Eu3+) composite for osteochondral defect regeneration and theranostics. Mater. Sci. Eng. C Mater. Biol. Appl..

[28] Zhang W., Liao S.S., Cui F.Z. (2003). Hierarchical Self-Assembly of Nano-Fibrils in Mineralized Collagen. Chem. Mater..

[29] Landis W.J., Song M.J., Leith A., McEwen L., McEwen B.F. (1993). Mineral and organic matrix interaction in normally calcifying tendon visualized in three dimensions by high-voltage electron microscopic tomography and graphic image reconstruction. J. Struct. Biol..

[30] Sun Y., Liu H., Sun X.-Y., Xia W., Deng C. (2021). In vitro and in vivo study on the osseointegration of magnesium and strontium ion with two different proportions of mineralized collagen and its mechanism. J. Biomater. Appl..

[31] Kane R.J., Roeder R.K. (2021). Effects of hydroxyapatite reinforcement on the architecture and mechanical properties of freeze-dried collagen scaffolds. J. Mech. Behav. Biomed. Mater..

[32] Chen Y.-C., Hsu P.-Y., Tuan W.-H., Chen C.-Y., Wu C.-J., Lai P.-L. (2021). Long-term in vitro degradation and in vivo evaluation of resorbable bioceramics. J. Mater. Sci. Mater. Med..

[33] Xing F., Chi Z., Yang R., Xu D., Cui J., Huang Y., Zhou C., Liu C. (2021). Chitin-hydroxyapatite-collagen composite scaffolds for bone regeneration. Int. J. Biol. Macromol..

[34] Fu X., Liu G., Halim A., Ju Y., Luo Q., Song G. (2019). Mesenchymal Stem Cell Migration and Tissue Repair. Cells.

[35] Chen X., Hu J., Huang Y., Li S., Li S., Wang M., Xia H., Li-Ling J., Xie H. (2020). Copper promotes the migration of bone marrow mesenchymal stem cells via Rnd3-dependent cytoskeleton remodeling. J. Cell. Physiol..

[36] Dewey M.J., Johnson E.M., Weisgerber D.W., Wheeler M.B., Harley B.A. (2019). Shape-fitting collagen-PLA composite promotes osteogenic differentiation of porcine adipose stem cells. J. Mech. Behav. Biomed. Mater..

[37] Mehrabani M.G., Karimian R., Mehramouz B., Rahimi M., Kafil H.S. (2018). Preparation of biocompatible and biodegradable silk fibroin/chitin/silver nanoparticles 3D scaffolds as a bandage for antimicrobial wound dressing. Int. J. Biol. Macromol..

[38] Rüdrich U., Lasgorceix M., Champion E., Pascaud-Mathieu P., Damia C., Chartier T., Brie J., Magnaudeix A. (2019). Pre-osteoblast cell colonization of porous silicon substituted hydroxyapatite bioceramics: Influence of microporosity and macropore design. Mater. Sci. Eng. C Mater. Biol. Appl..

[39] Kretlow J.D., Mikos A.G., Kengla C., Renteria E., Wivell C., Atala A., Yoo J.J., Lee S.J., Shin K., Acri T. (2007). Review: Mineralization of synthetic polymer scaffolds for bone tissue engineering. Tissue Eng..

[40] Hutmacher D.W., Schantz J.T., Lam C.X.F., Tan K.C., Lim T.C. (2007). State of the art and future directions of scaffold-based bone engineering from a biomaterials perspective. J. Tissue Eng. Regen. Med..

[41] Tajbakhsh S., Hajiali F. (2017). A comprehensive study on the fabrication and properties of biocomposites of poly(lactic acid)/ceramics for bone tissue engineering. Mater. Sci. Eng. C Mater. Biol. Appl..

