# Correction: Zhu et al. Mineralized Collagen/Polylactic Acid Composite Scaffolds for Load-Bearing Bone Regeneration in a Developmental Model. *Polymers* 2023, *15*, 4194

**DOI:** 10.3390/polym16020279

**Published:** 2024-01-19

**Authors:** Wenbo Zhu, Wenjing Li, Mengxuan Yao, Yan Wang, Wei Zhang, Chao Li, Xiumei Wang, Wei Chen, Hongzhi Lv

**Affiliations:** 1 Department of Orthopaedic Surgery, Hebei Medical University Third Hospital, No. 139 Ziqiang Road, Shijiazhuang 050051, China; 2 Key Laboratory of Biomechanics of Hebei Province, Orthopaedic Research Institution of Hebei Province, No. 139 Ziqiang Road, Shijiazhuang 050051, China; 3 National Health Commission Key Laboratory of Intelligent Orthopaedic Equipment, Hebei Medical University Third Hospital, No. 139 Ziqiang Road, Shijiazhuang 050051, China; 4 Department of Pathology, Hebei Medical University, No. 361 Zhongshan Road, Shijiazhuang 050017, China; 5 State Key Laboratory of New Ceramics and Fine Processing, School of Materials Science and Engineering, Tsinghua University, No. 30 Shuangqing Road, Beijing 100084, China

In the original publication [1], there was a mistake in Figure 6 as published. When we conducted this animal study, three sheep were operated on. When we prepared Figure 6, figure A was wrongly mixed up with figures B and C, which were from different sheep. The correct Figure 6 is listed below, with figure A, B, and C from the same sheep. The corrected Figure 6 appears below. The authors apologize for any inconvenience caused and state that the scientific conclusions are unaffected. This correction was approved by the Academic Editor. The original publication has also been updated.

## Figures and Tables

**Figure 6:**
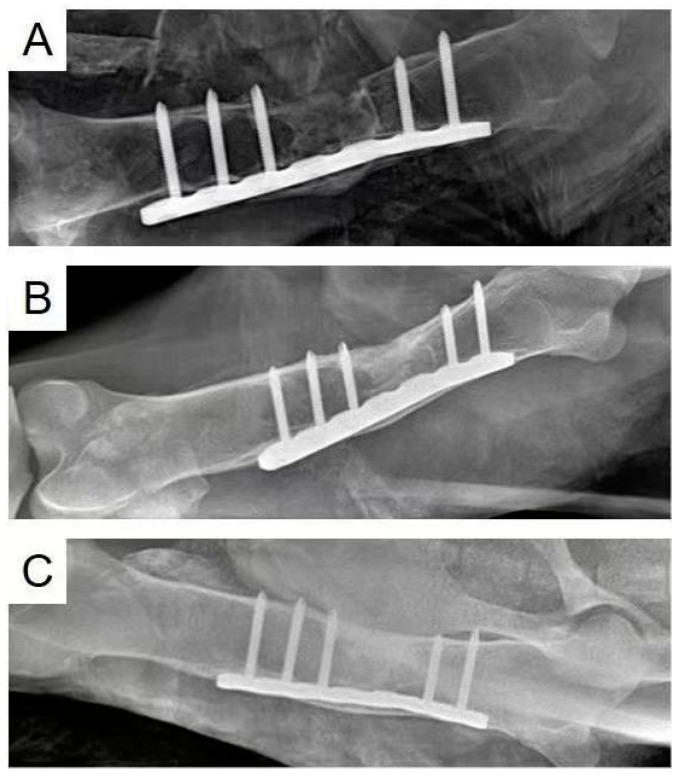
X-ray images of the femur defect at 1 (**A**), 3 (**B**), and 6 (**C**) months after the MC/PLA scaffold implantation in 3-month-old sheep.
